# A Novel Positioning System Based on Coverage Area Pruning in Wireless Sensor Networks

**DOI:** 10.3390/s18124469

**Published:** 2018-12-17

**Authors:** Shih-Chang Huang, Fu-Gong Li

**Affiliations:** Department of Computer Science and Information Engineering, National Formosa University, Yunlin 63201, Taiwan; darkjack6428@gmail.com

**Keywords:** range-free positioning, coverage area pruning, degree of irregularity, centroid point, wireless sensor networks

## Abstract

Wireless sensor networks are commonly applied in environmental monitoring applications. The crucial factor in such applications is to accurately retrieve the location of a monitoring event. Although many technologies have been proposed for target positioning, the devices used in such methods require better computational abilities or special hardware that is unsuitable for sensor networks with limited ability. Therefore, a range-free positioning algorithm, named coverage area pruning positioning system (CAPPS), is proposed in this study. First, the proposed CAPPS approach determines the area that includes the target approximately by using sensor nodes that can detect the target. Next, CAPPS uses sensor nodes that cannot detect the target to prune the area to improve positioning accuracy. The radio coverage variation is evaluated in a practical scenario, and a heuristic mechanism is proposed to reduce false positioning probability. Simulation results show that the size of the positioning area computed by CAPPS is smaller than that computed using distance vector hop, angle of arrival, and received signal strength indicator by approximately 98%, 97%, and 93%, respectively. In the radio variation scenario, the probability of determining an area excluding the target can be reduced from 50%–95% to 10%–30% by applying the proposed centroid point mechanism.

## 1. Introduction

Wireless sensor networks (WSNs) are commonly applied to all types of environmental monitoring, such as agricultural status monitoring [1], animal habitat observation [2,3], climate monitoring [4,5], and forest fire warning [6,7]. Essentially, a wireless sensor network consists of many sensor nodes that are tiny devices with simple computational abilities and specific sensing devices installed. Sensor nodes also include a wireless communication module for delivering data wirelessly to the data center, which is also named as the sink. Sensor nodes are spread throughout a region of interest (RoI). They are steady in their deployed locations and transmit local information to the sink through wireless propagation.

Determining the locations of target events accurately is a key factor to make these applications successful. Many object-positioning methods have been proposed, and these can be classified into two categories: range-based and range-free methods. Range-based methods require the distance between the target and each reference point to compute the position of a target. Here, the reference point is a node or device that can accurately obtain its positioning or coordinates. Owing to the location of the target is unknown, the real Euclidean distance between the target and reference point cannot be measured. Therefore, some methods have been proposed to obtain the estimated distance by inferring it from the received signal strength (RSS) [8,9,10,11,12], or signal propagation time [13,14,15]. Then, three reference points that are not collinear are selected. The location of each reference point is treated as the center of a circle, the radius of which gives the estimated distance to the target. Finally, the positioning problem is transformed to that of finding the intersection point of these three circles in a Euclidean plane. This intersection point gives the position of the target. However, the estimated distances inferred from the received RSS and signal propagation time are highly sensitive to shadowing effects, multipath effects, and multipath propagation in a wireless communication environment. A slight difference in the RSS or signal propagation time will introduce a significant error to the estimated distance.

For range-free methods, we usually require more than three reference points to locate the target. This characteristic is suitable for WSNs in which many quantity sensor nodes are deployed. Consequently, all sensor nodes deployed in the RoI must be aware of their positions, so that each of them can act as a reference point. As sensor nodes are densely deployed, they can cover the whole area of the RoI. Instead of attempting to find the estimated distances between the target and reference points, range-free methods collect information from the deployed sensor nodes to deduce the location of the target. The collected information can comprise the locations of the sensor nodes within the coverage area that can detect the target [16]. The information could also be the charging rates of sensor nodes [17] in a rechargeable wireless sensor network or the numbers of data propagation hops [18,19,20]. In addition, the signal variation that sensor nodes detect from the target [21,22] can also be used. A heuristic algorithm based on signal variation can be applied to estimate the possible location of the target. Without accurate distance information between the reference points and the target, range-free methods usually determine the target within a region instead of a specific point. Therefore, positioning accuracy is related to the size of the region. The smaller the obtained region, the higher the positioning accuracy will be.

Nowadays, well-known range-free positioning systems require reference nodes to either have specific hardware [17,23,24,25] or be able to compute the average one-hop distance [18,19,20], which is heavily dependent on the topology. When methods for which sensor nodes need to install specific hardware are applied to a sensor network, the manufacturing cost is increased. Furthermore, methods that are heavily dependent on the topology require rebuilding of the average one-hop distance. Such methods cannot provide high-accuracy positioning results. In addition, once the sensor nodes change their topology, the one-hop distance must be recomputed. 

Therefore, a novel range-free positioning algorithm is proposed in this study, named coverage area pruning positioning system (CAPPS). CAPPS does not require sensor nodes to install specific hardware, nor does it involve heavy dependence on the topology. Each sensor node in CAPPS must only provide feedback to the positioning station on whether it detects the target. Then, the positioning station is responsible for collecting the data of sensor nodes and computing the location of the target. Based on the Boolean results reported by the sensor nodes, the positioning station computes the intersection coverage areas of the sensor nodes. The description above describes the mechanism of the coverage elimination positioning system (CEPS) [26]. However, this only applies to target-detecting sensor nodes when locating the target. In our system, sensor nodes that cannot detect the target are also given location information about the target, and these sensor nodes can be involved in reducing the positioning area to improve the positioning accuracy. Therefore, CAPPS extends the CEPS mechanism. To reduce the computational overhead, CAPPS provides a mechanism to remove redundant sensor nodes. Then, the sensor nodes that cannot detect the target are involved to refine the positioning area. CAPPS can considerably reduce the computational overhead spent on locating the target. It is also robust against variations in the topology. In addition, the radio variation in a practical scenario is considered to evaluate the impact on the positioning accuracy. Simulation results show that CAPPS can locate the target within a smaller area compared to existing range-free positioning methods.

The remainder of this paper is organized as follows: Previous studies related to positioning technologies are reviewed in Section 2. Section 3 states the assumptions and presents the detailed algorithm of the proposed CAPPS approach. Section 4 presents the simulation results. In addition, the impact of radio variation is discussed in this section. Finally, conclusions are provided in Section 5.

## 2. Related Work

Anchor points, which can accurately obtain their own location information, are an essential element in a positioning method. The anchor points will be the reference points for determining the location of the positioning object. Essentially, current positioning technologies can be classified into two categories. The first comprises range-based methods that require the distances between anchor points and the positioning object to deduce the location of the positioning object. Because the location of the positioning object is unknown, this distance is estimated from other information, such as the RSS or signal propagation time. Well-known related range-based technologies include that using received signal strength indicator (RSSI) [8,9,10,11,12], time of arrival (ToA) [13], and time difference of arrival (TDoA) [14]. The second category is that of range-free methods that do not attempt to estimate the physical distance between an anchor point and positioning object. These methods use specific mechanisms to deduce the location of the positioning object, such as the incoming angle of the received signal, the radio variation of a signal, and the number of data propagation hops from the object to the anchor point. The number of hops consists of the number of intermediate devices through which data must pass between the object and anchor point. Well-known technologies in the category include angle of arrival (AoA) [23,24,25], time of charging (ToC) [17,27], point-in-triangulation (PiT), approximate point-in-triangulation (APiT) [21,22], and distance vector hop (DV-Hop) [18,19,20]. These methods usually do not need specific hardware modules, and they can therefore be applied to low-cost sensor nodes in WSNs. Consequently, the positioning accuracy in range-free methods is sacrificed slightly compared with that in range-based methods. Considering the application scenario, range-free methods are suitable for use in WSNs.

### 2.1. Range-Based Methods

A range-based method is triangulation location in which three anchor points are used. This method requires the distance from the target to each anchor point. The positioning problem is transformed into a problem of planar geometry. The position of each anchor point is the center of a circle, and the radius of this circle represents the distance between the anchor point and target. The intersection of these three circles gives the location of the target. The positioning accuracy is dependent on the measured distance from each anchor point to the target. Because the position of the target is unknown, this distance is usually measured using the RSS or signal propagation time. However, this indirectly measured information is highly sensitive to environmental factors. A slight inaccuracy may introduce a considerable error in the measured distance. In practical cases, the target is usually bounded within an area rather than at a point. The positioning accuracy is completely dependent on how the distance is measured. Three common distance measurement methods use RSSI, ToA, and TDoA, which will be introduced in the following.

The distance measured with the RSSI method [8,9] considers the RSS of the receiver. The signal decay model is given by (1), where *P_r_*(*d*) is the signal strength measured by the receiver, *P_t_* is the signal strength measured by the transmitter, *PL*(*d*_0_) gives the path loss parameters, $10n\log_{10}\frac{d}{d_{0}}$ is the distance decay coefficient, and *X_a_* is Gaussian noise:

(1) $$P_{r}\left(d\right)=\left\{Pt-PL\left(d_{0}\right)-10n\log_{10}\left[\frac{d}{d_{0}}\right]\right\}+X_{a}$$

To enhance the positioning accuracy, Tomic et al. proposed convex optimization approaches to address RSS-based cooperative localization problems in WSNs [10,11,12]. The maximum likelihood criterion is employed to formulate the localization problem. An appropriate convex relaxation technique leading to second-order cone programming is applied to overcome the non-convexity of the maximum likelihood problem. However, the radio signal is highly sensitive to the environmental factors, such as shadowing effects, multipath effects, and unstable magnetic fields. Therefore, the error of the measured distance is proportional to the physical distance between sender and receiver.

The ToA [13] method uses radio transmission speed and signal propagation time to measure the distance between the anchor point and target. Let *T*_0_ be the time instant at which the target sends the measurement message. This time instant *T*_0_ is also included within the measurement message. Any anchor point that receives this message at time instant *T*_1_ can compute the distance as (*T*_1_ − *T*_0_) × *V_e_*, where (*T*_1_ − *T*_0_) is the delivery time of the message and *V_e_* is the radio transmission speed, which is known to be 3 × 10^8^ m/s. ToA requires all devices involved in the positioning procedure to synchronize their system time beforehand. However, this is a challenging issue in the real world. To solve this problem, the procedure is modified slightly. The target sends a signal to the anchor, and then the anchor returns the message immediately back to the target. Two additional time instants are recorded in this modified procedure. These are the time instant *T*_2_ at which the anchor point sends the message and the time instant *T*_3_ at which the target receives the returned message from the anchor point. The turn-around time of the signal propagation between the target and the anchor point can be used to average the one-directional signal propagation. Therefore, the one-directional propagation time can be computed as ((*T*_3_ − *T*_0_)−(*T*_2_ − *T*_1_))/2. Consequently, as we know that the radio propagation speed is 3 × 10^8^ m/s, a minor variation in the average time still introduces a considerably large error in the measured distance.

The time synchronization problem of ToA is solved by TDoA [14,15]. Let *D*_1_ and *D*_2_ denote the measured distances from the target to anchor points 1 and 2, respectively. Then, |*D*_1_ − *D*_2_| is one local length. Similarly, anchor points 1 and 3 can generate another local length. Each local length can form a hyperbola, and the intersection of these two hyperbolas can provide the position of the target. TDoA does not require the system time synchronization between the target and anchor points. It is simple to implement and can enhance the location precision in non-line-of-sight environments.

### 2.2. Range-Free Methods

Rather than attempting to measure the physical distances between the anchor points and the target and applying triangulation location to locate the target, range-free methods employ other mechanisms to locate the target. One range-free method is AoA that requires every anchor point to install a directional antenna [23,24,25]. Each anchor point can identify the position of the target with the help of the directional antenna. A line equation on the Euclidean plane can be constructed by using the position of the anchor point and the direction of the received signal obtained from the directional antenna. Two anchor points with distinct positions can obtain two nonparallel lines, and the intersection point of these two lines will give the location of the target. Slavisa Tomic et al. integrated the RSS and AoA positioning methods with convex relaxation techniques [24] to improve positioning accuracy. Unfortunately, the direction provided by the directional antenna constitutes a fan-shaped area in practical scenarios. The target is bounded within the intersection region of these two fan-shaped areas. The positioning results degrade as the distance from the target to the anchor point increases, because the size of this intersection region is proportional to the distance.

The ToC [17,27] method is applied in wireless rechargeable sensor networks. Every anchor point installs a wireless charging component. When the target is close to the anchor point, the target can achieve a high charging efficiency. Conversely, the target has a low charging rate if it is far away from the anchor point. Based on this characteristic, we can deduce the possible distance between the anchor point and the target. This method requires more than two anchor points to position the target. Chang et al. proposed a method integrating AoA and ToC [27] to position the target using one anchor point.

The PiT [21,22] method uses radio signal attenuation characteristics to position the target. The target is assumed to be moveable. Three random anchor points form a triangle, and whether the target is enclosed in this triangular area is determined. When the target moves, the distance from its current location to each anchor point will change. When the distance changes, the RSS received by each anchor point will also vary. If the current location of the target is within the triangle, then this moving behavior will cause the RSS values of two anchor points to increase and that of one anchor point to decrease. If all the RSS values of all three anchor points increase or decrease, then the target must be outside the triangle. This method is simple, and requires no distance information. However, it requires a movable and controllable target. It cannot be applied in a scenario in which the position of the target is unknown. 

The APiT [21,22] method is similar to PiT but applies to unmovable targets. When a neighbor node has a distinct location from the target, the RSSI value of this neighbor node can be treated as that of the target stays at this location. Consequently, the relative location information must be known beforehand for all neighbor nodes in the network. A previous study [28] has shown that APiT requires at least 24 neighbor nodes to achieve a positioning error lower than 5%. 

DV-Hop [18,19,20] is a range-free positioning method that still attempts to estimate the distances between the target and anchor points. The distance is estimated from the number of signal propagation hops. DV-Hop still requires three anchor points to locate the target. Let the three anchor points be denoted by A, B, and C. The anchor points send a message to each other, and the numbers of propagation hops are recorded. By using the physical distance between two anchor points and the number of propagation hops from one anchor point to another, the average physical distance of one propagation hop can be computed. The average distance of one propagation hop, denoted by *h_avg_*, can be computed as in (2):

(2) $$h_{avg}=\frac{\left|\bar{AB}\right|+\left|\bar{AC}\right|}{H\left(AB\right)+H\left(AC\right)}$$

Here, $\left|\bar{AB}\right|$ and $\left|\bar{AC}\right|$ denote the physical distances between two anchor points and can be computed from their coordinates, and *H*(*AB*) and *H*(*AC*) are the numbers of hops between the two anchor points. Then, the target sends a message to each anchor point, and the numbers of propagation hops are recorded. The distance is determined from the obtained results by multiplying the number of propagation hops by the average distance of a single hop.

The distance estimation mechanism in DV-Hop depends strongly on the deployment topology. A significant error may be introduced when the shortest routing path from the target to an anchor point is not close to the distance of the direct link from the target to that anchor point. This occurs when the sensor nodes are not uniformly deployed or the deployment density is not sufficiently high. Therefore, Yuan et al. proposed a mechanism to improve the accuracy of the average one-hop distance [29]. The equation for refining *h_avg_* is given in (3):

(3) $$h_{avg}^{*}=h_{avg}-\frac{E_{A}}{H\left(AB\right)+H\left(AC\right)},$$

where *E_A_* is computed as in (4):

(4) $$E_{A}=\left(\left|\bar{AB}\right|+\left(h_{avg}\times H\left(AB\right)\right)\right)+\left(\left|\bar{AC}\right|-\left(h_{avg}\times H\left(AC\right)\right)\right)$$

The range-free methods described above that require sensor nodes to install special hardware are unsuitable for low-cost sensor nodes in a general sensor network. Furthermore, the methods that are strongly related to topology cannot provide an acceptable positioning accuracy. Therefore, CAPPS is proposed in this study. CAPPS does not require special hardware; it uses homogeneous sensor nodes with common omni-directional antenna. CAPPS employs sensor nodes that detect the target to determine the possible area of its location. Then, the sensor nodes that cannot detect the target are employed to refine the size of the positioning area, to enhance the positioning accuracy.

## 3. Coverage Area Pruning Positioning System

The mechanism of the proposed CAPPS is described in this section in detail. First, the preliminaries and assumptions are presented. Then, the methodologies using the sensor nodes that do and do not detect the target to determine and prune the positioning area, respectively, are presented. Finally, the impact of signal irregularity is considered, and a mechanism named centroid point (*CP*) is proposed to moderate this influence.

### 3.1. Preliminary and Assumptions

To apply CAPPS, the following assumptions are required. The deployed sensor nodes are homogeneous and uniformly spread within the RoI. The maximum radio range of every sensor node is *R*. The entire RoI can be covered by the radio signals of the deployed sensor nodes. Therefore, the target can be guaranteed to be detected by at least one sensor node in the RoI. A self-deploying method [30] may be applied to achieve this assumption.

All deployed sensor nodes are stationary. Each of them can precisely retrieve its current location. Therefore, every sensor node can act as an anchor point, like the sensor nodes in the PiT and APiT methods. The computational ability of each sensor node is sacrificed to prolong the operational duration. Thus, each sensor node can send data to the *positioning station* to locate the target. A positioning station is employed to coordinate the operations of the sensor nodes. When a sensor node detects the target, it automatically notifies the positioning station immediately.

### 3.2. Locating the Positioning Area with Target-Detecting Sensor Nodes

The CAPPS method is an extension of a previous method named CEPS [26]. When the target appears in the RoI, the sensor nodes that detect the target transmit their detection message to the positioning server. Let *Ω* denote the set of these sensor nodes. The positioning problem is transformed to that of finding the intersection area of the sensor nodes in *Ω*. For each sensor node *S_i_* ∈ *Ω*, *S_i_* is represented by a circle *C_i_*, as shown in (5):

(5) $$C_{i}:{\left(x-S_{i.x}\right)}^{2}+{\left(y-S_{i.y}\right)}^{2}=R^{2},i=1,2,3,\ldots ,\left|\Omega \right|$$

Here, *S_i.x_* and *S_i.y_* in (5) denote the x- and y-coordinates, respectively, of *S_i_*. The target will be enclosed within the area covered by all sensor nodes in *Ω*. This can be represented as ${⋃}_{i=1}^{\left|\Omega \right|}C_{i}$. In the following, the *positioning area* is used to represent the possible region that includes the target. The smaller the positioning area, the higher the positioning accuracy will be.

**Figure 1:**
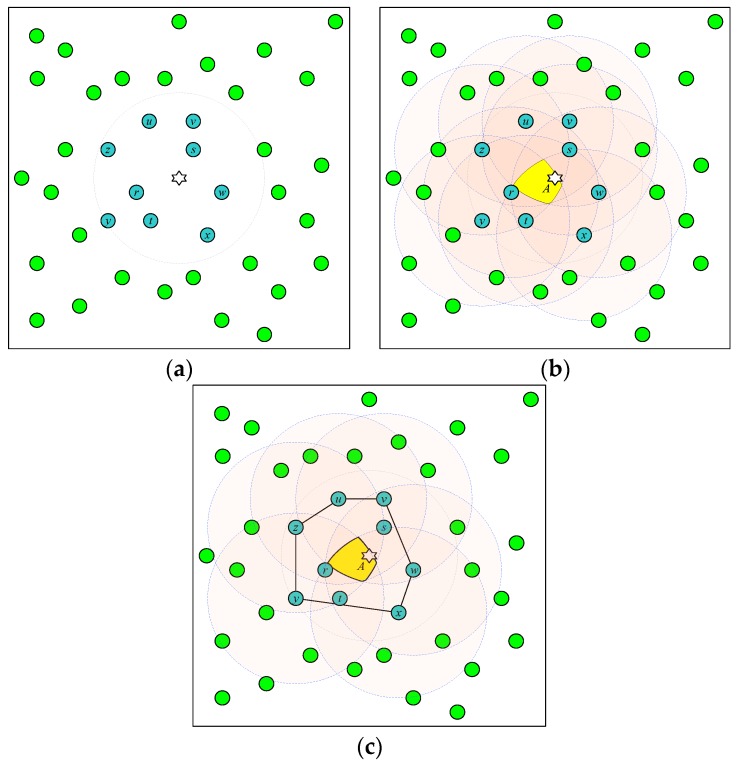
Example of locating the target using target-detecting sensor nodes. (**a**) The target represented as a hexagonal star and the sensor nodes in the set *Ω* = {*r*, *s*, *t*, *u*, *v*, *w*, *x*, *y*, *z*} represented as alphabetical circles. (**b**) The positioning area obtained from the intersection area of the radio coverage of the sensor nodes in the set *Ω*. (**c**). The positioning area obtained from the intersection area of the radio coverage of the sensor nodes in the set *Ω_c_* = {*u*, *v*, *w*, *x*, *y*, *z*}. The result is the same as that using the set *Ω*.

A simple example is presented in Figure 1. In Figure 1a, sensor nodes {*r*, *s*, *t*, *u*, *v*, *w*, *x*, *y*, *z*} ∈ *Ω* detect the signal of the target. Each sensor node in *Ω* is treated as a vertex on the Euclidean plane. Every vertex represents the center of a circle with the radius set to the radio range *R*. By computing the intersection area of these circles, we can obtain the area *A* shown in Figure 1b that includes the target.

This procedure contains redundant computations. For example, the coverage areas of {*r*, *s*, *t*} are completely enclosed by those of the other sensor nodes in *Ω*. Removing these sensor nodes does not influence the size of the positioning area. This observation implies the following two characteristics. First, the sensor nodes that detect the target but are far away from it contribute efficiently to the positioning area. Second, the sensor nodes *S_i_* in *Ω* with no contribution to the positioning area are usually close to the target, such that their coverage area is completely overlapping with other sensor nodes. These two characteristics imply that the vertices located at the outer bound of the signal area of the target are sufficient. Fortunately, the convex hull algorithm can be applied to determine the sensor nodes located in the outer area.

The convex hull problem is defined as follows. Considering a set of points in the Euclidean plane, a set of points is defined to be convex if it contains the line segments connecting each pair of its points. The convex hull of a given set Z is the minimal convex set containing Z, and the intersection of all convex sets contains Z. In addition, the set of all convex combinations of points in Z represents the union of all simplexes with vertices in Z. Therefore, the sensor nodes that constitute the convex hull of the set Z must be the nodes located at the outer boundary of the points in the set Z.

The set of vertices comprising the convex hull of the set *Ω*, denoted as *Ω_c_*, can be used to compute the positioning area of the target. As shown in Figure 1c, the sensor nodes {*u*, *v*, *w*, *x*, *y*, *z*} ∈ *Ω_c_* comprise the vertices used to compute the intersection area. The CEPS method employing the convex hull to compute the positioning area is denoted by C-CEPS in the simulation section. 

### 3.3. Pruning the Positioning Area with Non-Target-Detecting Sensor Nodes

**Figure 2:**
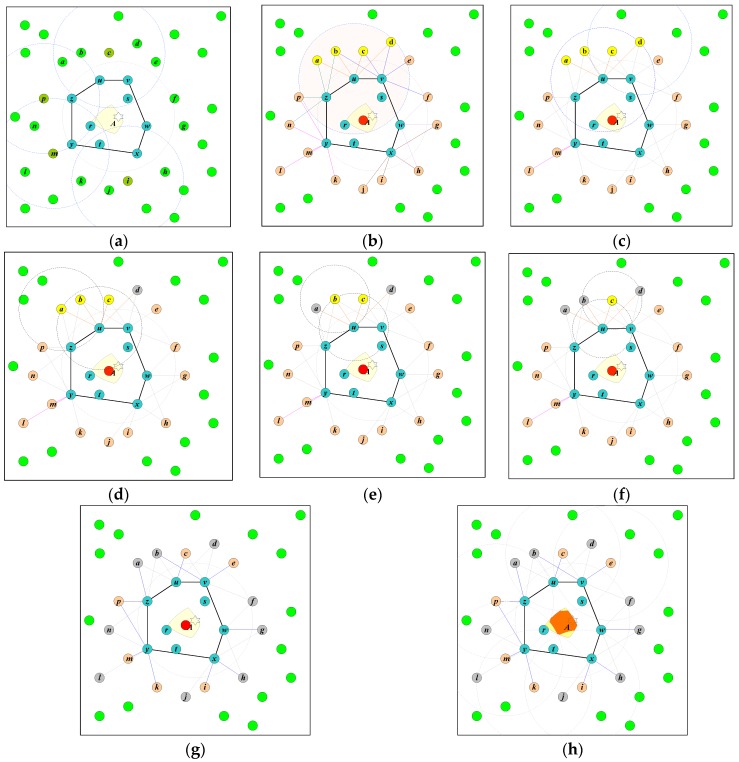
Illustrating the positioning procedure of CAPPS. In this example, *Ω* = {*r*, *s*, *t*, *u*, *v*, *w*, *x*, *y*, *z*} and *Ω_c_* = {*u*, *v*, *w*, *x*, *y*, *z*}. (**a**) The coverage areas of the non-target-detecting sensor nodes {*c*, *i*, *m*, *p*} overlap with the positioning area. (**b**) The sensor nodes in the set *Ω* are used to compute *T_g_*, illustrated by the circle located at the positioning area. The one-hop neighbors of the sensor node *u* are identified, and the sorted sequence is stored in the set $\Pi_{u}^{*}$ = {*d*, *a*, *b*, *c*}. (**c**) Verify the first sensor nodes *d* of the set $\Pi_{u}^{*}$. The sensor node *c* is enclosed within the intersection area *A_ud_*. Thus, the sensor node *d* is removed from the set $\Pi_{u}^{*}$. (**d**–**f**). Verify the other sensor nodes *a*, *b*, and *c* in the set $\Pi_{u}^{*}$. (**g**) The set of remaining sensor nodes for pruning the positioning area *Γ* = {*c*, *e*, *i*, *k*, *m*, *p*}. (**h**) The pruned positioning area obtained by applying the sensor nodes in the set *Γ*.

When a sensor node cannot detect the target, this indicates that the target is not within its coverage area. If the coverage area of the sensor node overlaps with the positioning area obtained by the sensor nodes that detect the signal of the target, then the positioning accuracy can be further improved. As shown in Figure 2a, the sensor nodes {*c*, *i*, *m*, *p*} cannot detect the target, but their coverage areas overlap with the positioning area. Therefore, the positioning area can be further pruned to enhance the positioning accuracy.

The simplest manner of pruning the positioning area is to consider all sensor nodes that cannot detect the signal of the target. However, this method is inefficient, especially when there are many sensor nodes in the network. To find the sensor nodes that cannot detect the signal of the target but can prune the positioning area, the following mechanism is employed. First, the centroid of the coordinates of the sensor nodes in the set *Ω*, denoted as *T_g_*, is computed. In general, the centroid *T_g_* is close to the location of the target if the sensor nodes are uniformly deployed. The coordinates of centroid *T_g_* are computed using (6):

(6) $$T_{g}\left(x,y\right)=\left(\frac{\sum_{i=1}^{\left|\Omega \right|}S_{i.x}}{\left|\Omega \right|},\frac{\sum_{i=1}^{\left|\Omega \right|}S_{i.y}}{\left|\Omega \right|}\right)$$

Next, the one-hop neighbors of each sensor node *β_i_* ∈ *Ω_c_* are retrieved. These one-hop neighbors must be included in *Π* = {∪*Π_i_* | *i* = 1, 2, 3, …, |*Ω_c_*|}, where *Π_i_* is the set of one-hop neighbors of the sensor node *β_i_*. The sensor nodes in each *Π_i_* are sorted in descending order according to their distance to *T_g_*. Let the sorted set of *Π_i_* be denoted by $\Pi_{i}^{*}$. The following mechanism is applied to remove the sensor nodes in *Π* that cannot prune the positioning area. Sensor nodes $S_{k}^{i}\in \Pi_{i}^{*}$ are sequentially selected from *k* = 1 to |$\Pi_{i}^{*}$|, and $D*=|\bar{\beta_{i}S_{k}^{i}}|$ is computed. The coordinates of each of *β_i_* and *S^i^_k_* are employed to define the center of a circle with the radius set to D*. If there is any sensor node $S_{w}\in \Pi$ and $S_{w}\notin \{\beta_{i},S_{k}^{i}\}$ enclosed within the intersection area of these two circles, this implies that *S_w_* can contribute more efficiently to the pruning area than *S^i^_k_*. This is because *S_w_* is closer to the target, and its coverage area can completely cover the pruning area of *S^i^_k_*. Therefore, *S^i^_k_* is removed from the set $\Pi_{i}^{*}$.

This procedure continues until all sensor nodes in $\Pi_{i}^{*}$ are processed. The remaining sensor nodes left in $\Pi_{i}^{*}$ are used to prune the positioning area. When all $\Pi_{i}^{*}$ are processed, the remaining sensor nodes in each $\Pi_{i}^{*}$ can be represented as the set *Γ*, computed as in (7):

(7) $$\Gamma ={\overset{\left|\Omega \right|}{\underset{i=1}{⋃}}\Pi }_{i}^{*}$$

The sensor nodes in *Γ* are selected to prune the positioning area.

Figure 2 presents a simple example to illustrate this operation. We have sets *Ω* = {*r*, *s*, *t*, *u*, *v*, *w*, *x*, *y*, *z*} and *Ω_c_* = {*u*, *v*, *w*, *x*, *y*, *z*}. The set *Ω* is employed to compute *T_g_* according to (6). Its location is represented by the circle located in the positioning area shown in Figure 2b. For the sensor nodes in *Ω_c_*, their one-hop neighbors are *Π_u_* = {*a*, *b*, *c*, *d*}, *Π_v_* = {*b*, *c*, *d*, *e*, *f*}, *Π_w_* = {*f*, *g*, *h*}, *Π_x_* = {*h*, *i*, *j*}, *Π_y_* = {*k*, *l*, *m*, *n*}, and *Π_z_* = {*a*, *b*, *p*}. Therefore, *Π* = {*a*, *b*, *c*, *d*, *e*, *f*, *g*, *h*, *i*, *j*, *k*, *l*, *m*, *n*, *p*}.

For the sensor node *u*, its sorted one-hop neighbors are stored in the set $\Pi_{u}^{*}$ = {*d*, *a*, *b*, *c*}. Let *A_ud_* be the intersection area of the two circles centered at sensor nodes *u* and *d*, each with radius $D^{*}=|\bar{ud}|$. There is a sensor node *c* enclosed in *A_ud_*, as shown in Figure 2c. Therefore, the sensor node *d* is removed from the set $\Pi_{u}^{*}$. Next, the intersection area *A_ua_* of the circles centered at sensor nodes *u* and *a*, each with radius $D^{*}=|\bar{ua}|$, is evaluated as shown in Figure 2d. The sensor node *a* is removed because the sensor node b lies within the area *A_ua_*. The sensor node *b* is also removed for the same reason, as shown in Figure 2e. Finally, the sensor node *c* is checked, and no sensor node is found enclosed in the area *A_uc_*. Thus, the sensor node *c* is kept in the set $\Pi_{u}^{*}$ for pruning the positioning area, as shown in Figure 2f.

The same procedure is applied to all *β_i_* in the set *Ω_c_* to retrieve all $\Pi_{i}^{*}|\forall \beta_{i}\in \Omega_{c}$. The final set of sensor nodes for pruning the positioning area is *Γ* = {*c*, *e*, *i*, *k*, *m*, *p*}, as shown in Figure 2g. The positioning area pruned by the sensor nodes in the set *Γ* is shown in Figure 2h. There remain two redundant sensor nodes in the set *Γ*: the sensor nodes *e* and *k*. However, this procedure efficiently reduces the number of redundant sensor nodes significantly. The algorithm of CAPPS is presented in Algorithm 1.

**Algorithm 1.** The algorithm of CAPPS.
R: the maximum sensing range of a sensor node*S_i_*: the *i*th sensor node*T*: the target node*Ω*: the set of sensor nodes that can receive a signal from *T**C_i_*: the circle function of node *i*//InitialFor *S_i_* | *i* = 1, 2, 3, …., N { If *S_i_* detects the signal of *T*  Add *S_i_* to *Ω* Else   Add *S_i_* to ΨCompute the convex hull from the nodes in *Ω* and store the vertices in *Ω_c_*.Compute the intersection area of the nodes *S_i_* ∈ *Ω_c_*, denoted as *A_pos_*. For *S_i_* ∈ *Ω_c_* | *i* = 1, 2, 3, …,|*Ω_c_*| {   Find the one-hop neighbors and store in each *Π_i_*. }Set *Π* = ∪*Π_i_* | *i* = 1, 2, 3, …, |*Ω_c_*|}Compute the centroid point *V* of the sensor nodes in *Ω*∪*Π*For each *Π_i_*, *i* = 1, 2, 3, …,|*Ω_c_*| { Sort the sensor nodes *S_j_* in descending order according to length of |*VS_j_*| } For each *Π_i_*, *i* = 1, 2, 3, …,|*Ω_c_*| {  For each *S_j_* ∈ *Π_i_*, *j* = 1, 2, 3, ….,|*Π_i_*| {   Get two circles *C_i_* and *C_j_* that are centered at *S_i_* and *S_j_*, respectively, with radius set to |*S_i_S_j_*|.   If any node *S_w_* ∈ *Π*, *S_w_*, {*S_i_*, *S_j_*} is in the intersection area of *C_i_* and *C_j_*    Remove *S_j_* from *Π_i_* }   Denote the processed *Π_i_* as $\Pi_{i}^{*}$} Set Π^*^ = {$\Pi_{1}^{*}$, $\Pi_{2}^{*}$, $\Pi_{3}^{*}$, …, $\Pi_{\left|\Omega_{c}\right|}^{*}$}For each *S_k_* ∈ $\Pi_{i}^{*}$, {  Get Cov(*C_k_*), the coverage area of circles *C_k_* centered at *S_k_* with radius R.   *A_pos_* = *A_pos_* – *A_pos_* ∩ Cov(*C_k_*)}


### 3.4. Signal Irregularity and the Centroid Point Mechanism

**Figure 3:**
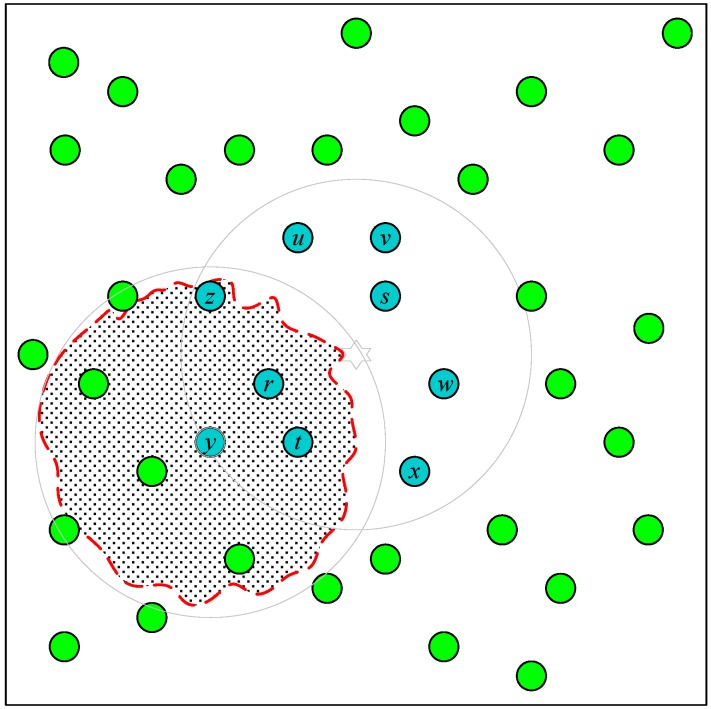
Signal irregularity in CAPPS.

Whether or not a sensor node can detect the signal of the target is the critical factor for CAPPS. However, radio signals are sensitive to environmental factors, such as temperature, obstacles, and magnetism. The practical radio coverage of a sensor node will be similar to the irregular dashed-line circle shown in Figure 3 rather than a perfect circle. When a sensor node cannot detect the signal of the target, the target may be outside of the coverage area of the sensor node, or it could be inside with environmental factors preventing the sensor node from detecting it. The latter case will lead CAPPS to reaching the wrong decision by employing the sensor node to prune the positioning area. This causes the target to be excluded from the possible positioning area.

As shown in Figure 3, the signal coverage area of the node *y* should contain the target. However, the signal irregularity makes it unable to detect the target. When the node *y* is selected to prune the positioning area, the target will be expelled from the positioning area. Let the perfect coverage area of the sensor node *y* be denoted by *P*, and the practical area represented by the shadowed area in Figure 3 be *Q*. The *uncertainty region* (*uReg*) is defined as *P* − *Q*. The CP mechanism is proposed to reduce the probability of pruning the target to be outside of the positioning area under an irregular signal scenario. The proposed CP mechanism employs the sensor nodes in the set *Ω* ∪ *Π* to compute the centroid point *P_v_*. We know that if most of the deployed sensor nodes in the set *Ω* are concentrated at one side of the target, these clustered sensor nodes will have many common one-hop neighbors. Conversely, the sensor nodes of *Ω* on the other side will have fewer one-hop neighbors. Therefore, if the sensor nodes in the set *Π* are involved in computing the centroid point, the less common one-hop neighbors will help the sensor nodes of the set *Ω* that are not on the clustered side to pull the centroid point in their direction. Therefore, integrating the sensor nodes in the sets *Ω* and *Π* can reduce the distance bias between *P_v_* and the real location of the target. All sensor nodes *S_i_* ∈ *Π* compute the distance $\left|\bar{P_{v}S_{i}}\right|$. If $\left|\bar{P_{v}S_{i}}\right|\leq R+\varepsilon$, then *S_i_* is removed from the set *Π*. Therefore, *S_i_* will not participate in pruning the positioning area. Here, *ε* is computed from the positioning area obtained from the target-detecting sensor nodes. Let this area be of size *A_CE_* obtained by applying the mechanism described in Section 3.2. Then, *ε* will be $\sqrt{\frac{A_{CE}}{\pi }}$. It should be noted that the probability of expelling the practical location of the target from the positioning area can be reduced. Consequently, the positioning accuracy will be sacrificed when we remove the sensor nodes with uncertain locations. The results are evaluated in the simulation section.

## 4. Simulation Results

Next, the proposed method is simulated and compared with existing methods. The environmental setup and simulation parameters are defined in the first subsection, and the numerical results are given in the second. The evaluation metric includes the impacts of the number of sensor nodes and the radio range on positioning accuracy. The computation time of the proposed method is also evaluated. By considering a practical case, the impact signal irregularity on the positioning accuracy is also discussed. All simulation results are the average over 2000 random deployment results.

### 4.1. Environment Setup

This section presents the evaluation results. Sensor nodes are randomly deployed over a 100 × 100 m^2^ rectangular area containing no obstacles. Each of these can obtain its accurate location information. The numbers of sensor nodes simulated in this study are {30, 35, 40, 45, 50, 55, 60, 65}. The simulated radio range includes {15, 20, 25, 30} m. The proposed CAPPS system is implemented via the Java programming language. The AOA, DV-Hop, and RSSI positioning methods are also implemented for comparison. In the AoA method, two sensor nodes are employed to locate the target. The angle accuracy is 15° (±7.5°). For the RSSI method, the error rate in converting the received signal strength to the distance is 15%. Three sensor nodes are employed as the anchor points for applying the triangulation location. The related parameters for these methods are listed in Table 1.

### 4.2. Numerical Results

**Figure 4:**
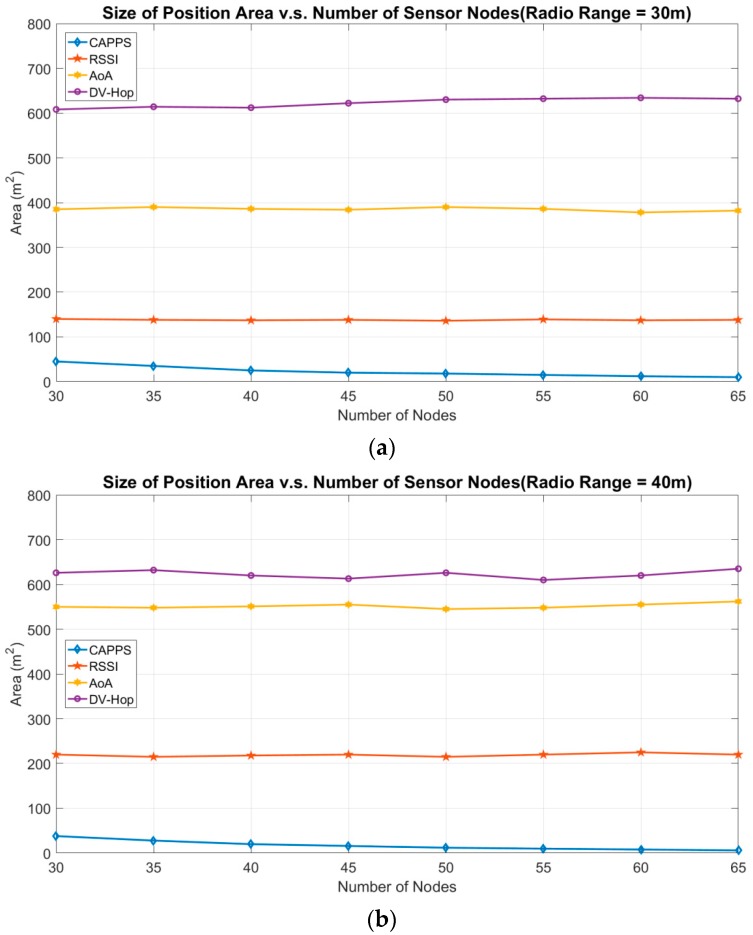
Size of position area vs. number of deployed sensor nodes. (**a**) The radio range of the sensor nodes in the experiment is set to 30 m. (**b**) The radio range of the sensor nodes in the experiment is set to 40 m.

Figure 4 depicts the positioning area sizes for RSSI, AoA, DV-Hop, and CAPPS with different numbers of sensor nodes. In Figure 4a, the radio range of each sensor node is set to 30 m. To display the results clearly, the numerical results depicted in Figure 4a are listed in Table 2. When the number of sensor nodes increases, the size of the positioning area for DV-Hop fluctuates between 608 m^2^ and 636 m^2^. Increasing the number of sensor nodes does not explicitly influence the numbers of propagation hops from the target to the anchor points. This is because the sensor nodes are uniformly deployed, and the density of sensor nodes is high enough to cover the entire RoI. The AoA and RSSI methods require a constant number of anchor points to position the target. Thus, increasing the number of sensor nodes does not introduce explicit variation in the positioning area. The size of the positioning area fluctuates within 378–390 m^2^ and 136–140 m^2^ respectively for the two methods. 

For CAPPS, sensor nodes with a higher deployment density can provide more chances to prune the positioning area. Thus, when the number of sensor nodes is 65, the size of the positioning area is 10 m^2^. Compared with the case with 30 sensor nodes, where the size of the positioning area is 47 m^2^, the improvement is approximately 79%. In the case with 65 sensor nodes, the positioning area of CAPPS is smaller than those of RSSI, AoA, and DV-Hop by approximately 93%, 97%, and 98%, respectively.

In Figure 4b, when the radio range of the sensor node increases to 40 m, the number of propagation hops from the target to each anchor point in the DV-Hop method decreases. Therefore, reducing the number of propagation hops can reduce the distance error for one hop, so that the size of the positioning area decreases. For the AoA method, the positioning area explicitly increases as the number of sensor nodes increases. We know that the positioning area in the AoA method is determined by two fan sectors obtained from two different sensor nodes. Therefore, the area of a sector increases exponentially when the radio range increases. The size of the positioning area with a radio range of 40 m is considerably larger than that for 30 m. The RSSI method exhibits a similar trend to the AoA method. Increasing the radio range will contribute to a larger distance estimation error, thereby increasing the positioning area. In the CAPPS method, increasing the radio range can extend the coverage area of each sensor node. Thus, each sensor node has a better opportunity to prune the positioning area; therefore, the size of the positioning area is reduced. As shown in Figure 4a,b, the size of the positioning area is between 47 m^2^ and 10 m^2^ with the radio range set to 30 m and is between 38 m^2^ and 6 m^2^ for a radio range of 40 m.

**Figure 5:**
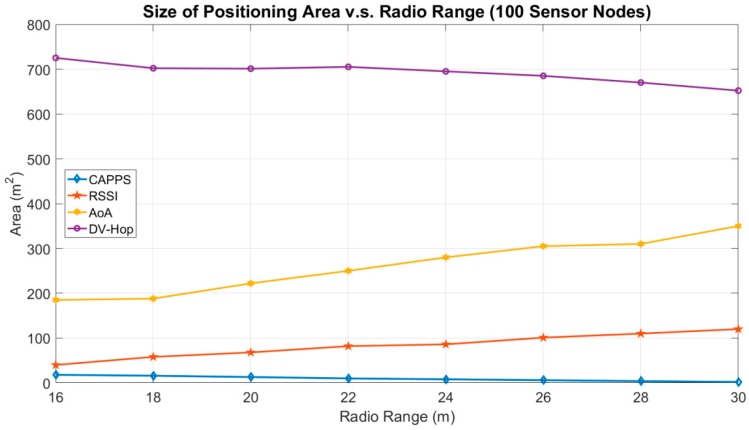
Size of positioning area vs. radio range.

Figure 5 illustrates the influence of the radio range on the size of the positioning area. To guarantee that the entire RoI can be covered by the sensor nodes, the number of sensor nodes in this experiment is set to 100. The size of the positioning area for DV-Hop decreases as the radio range increases. Then, the positioning area size fluctuates between 652 m^2^ and 725 m^2^. When a short radio range is employed, the average number of propagation hops computed in DV-Hop increases. More propagation hops imply more chances to introduce distance errors, because each hop may contribute to some distance error. Therefore, the size of the positioning area increases when a shorter radio range is employed. Conversely, when the radio range increases, the number of propagation hops decreases, and thereby the size of the positioning area decreases.

For the AoA method, the area of a sector increases exponentially as the radio range increases. Therefore, the size of the positioning area increases when the radio range increases. For the RSSI method, a larger distance estimation error is introduced as the radio range increases and in turn the size of the positioning area increases. This is similar to the results shown in Figure 4, where the positioning area size increases along with the radio range.

For CAPPS, deploying more sensor nodes can counteract the drawback of a short radio range. When the radio range is 16 m, the positioning area is approximately 18 m^2^. For a range of 30 m, the area is approximately 2 m^2^. These results are superior to those for ranges of 10 m^2^ and 6 m^2^ with 65 sensor nodes, as shown in Figure 4a,b, respectively. Therefore, increasing radio range can reduce the size of positioning area. This implies that the positioning accuracy can be enhanced when the radio range of the sensor nodes is increased.

**Figure 6:**
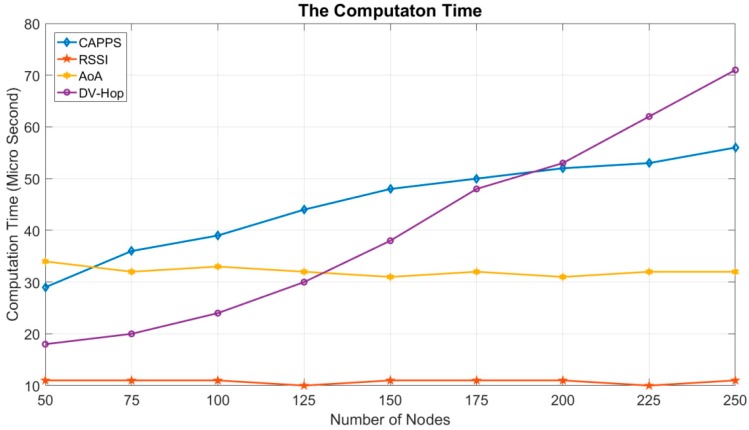
The computation time.

Figure 6 compares the computation times for each method. The radio range in this experiment is 30 m, and the number of deployed sensor nodes ranges from 50 to 250. The platform to perform these methods is equipped with an Intel I7-6700 CPU (3.4 GHz), and the memory is DDR4 2133 8 GB RAM. The graphics card is NVIDIA GeForce GT 430. The simulation results are averaged over 100 different deployment topologies. In this figure, the execution times of the AoA and RSSI methods do not increase when the number of sensor nodes increases. This is because the number of sensor nodes used in computing the positioning area in both methods is constant. The execution times of both DV-Hop and CAPPS increase as the number of sensor nodes increases.

In the DV-Hop method, each sensor node must compute its own average propagation hops to the anchor points. This requires less computation time when the network has fewer sensor nodes. When the number of sensor nodes increases, the computation time increases exponentially. In the CAPPS method, the computation overhead can be considerably reduced by first computing the one-hop neighbors of the sensor nodes in *Ω_c_*. When the number of nodes is greater than 200, the computation time of CAPPS becomes lower than that of DV-Hop.

**Figure 7:**
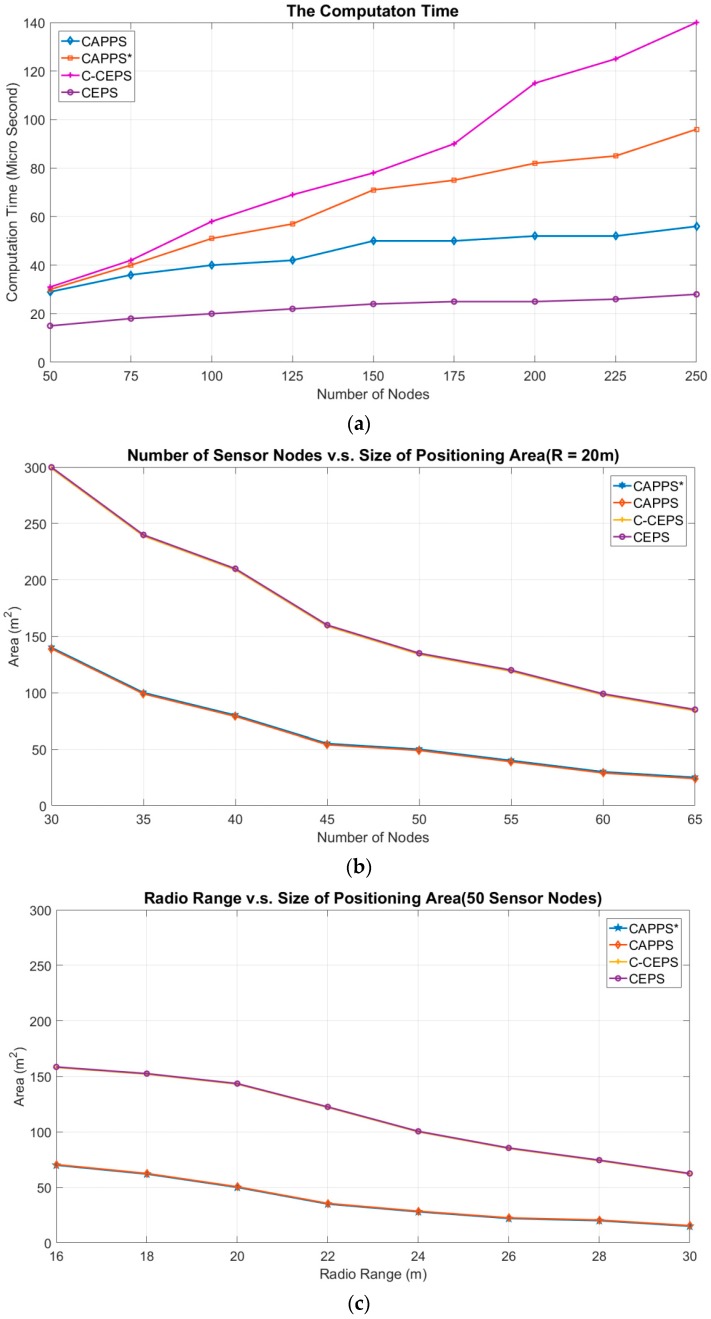
Comparison of CEPS, C-CEPS, CAPPS* and CAPPS. (**a**) The computation times of the CAPPS, CAPPS*, CEPS, and C-CEPS methods. (**b**) The impact of the number of deployed sensor nodes on the positioning area. The radio range in this experiment is 20 m. (**c**) The impact of the radio range on the positioning area. The number of sensor nodes in this experiment is 50.

In Figure 7a, the original CEPS method, C-CEPS, CAPPS*, and CAPPS are compared. The radio range of the sensor nodes in this figure is set to 30 m. The CAPPS* is the same as the CAPPS but using all one-hop neighboring target-undetected sensor nodes of the nodes in set *Ω_c_* to prune the positioning area. In our simulation, the size of the positioning area is computed by counting the pixels, which are included in the positioning area. The CAPPS method requires double the computation time of that of C-CEPS, because CAPPS must compute the pruning area for the sensor nodes that cannot detect the target. The computation time of CAPPS is lower than that of the original CEPS method, because many redundant sensor nodes are discarded in the positioning procedure. Without filtering the one-hop neighbors of the nodes in set *Ω_c_*, the CAPPS* spends more times on counting the number of pixels in the coverage area of each sensor node. Therefore, the computation time of CAPPS* is worse than that of the CAPPS. In addition, as the number of deployed sensor nodes grows, the computation overhead increases quickly. In the case of 250 deployed sensor nodes, the computation time is about 1.7 times higher than that of the CAPPS.

Figure 7b,c is the impacts of deployment density and radio range on the size of the positioning area. In Figure 7b, the radio range is set to 20 m, and the number of sensor nodes in Figure 7c is set to 50. For all methods, more sensor nodes can be involved in locating the target when the density of the deployed sensor nodes increases. Therefore, the size of the positioning area decreases when the number of sensor nodes increases. Both the CEPS and C-CEPS methods yield the same size for the positioning area, because C-CEPS only reduces the computation time by removing redundant sensor nodes. The CAPPS method yields a significant reduction in the size of the positioning area. The improvement ratio is approximately between 56% and 76%. This proves that by applying the sensor nodes that cannot detect the target to prune the positioning area, CAPPS can efficiently enhance the positioning accuracy. The CAPPS* and CAPPS yield the same size for the positioning area. CAPPS reduces the computation time by removing redundant one-hop neighboring sensor nodes.

**Figure 8:**
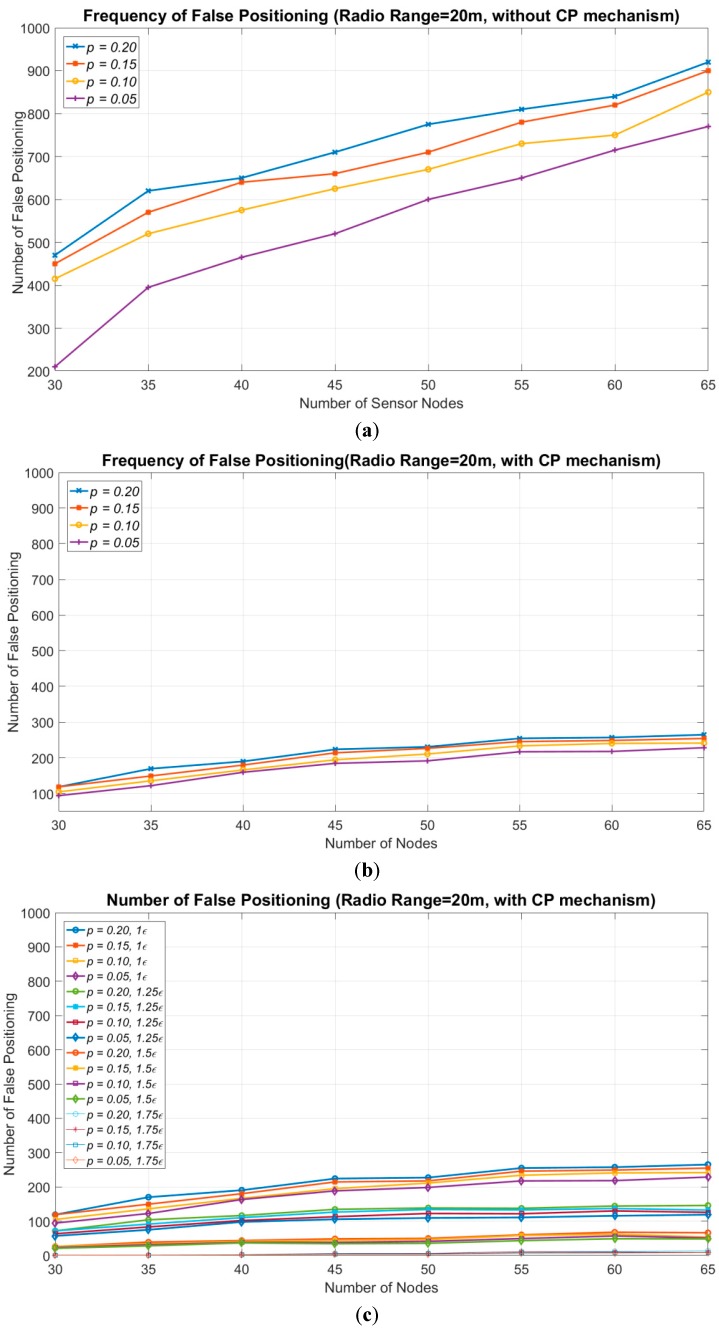
The frequency of false positioning vs. the number of sensor nodes. (**a**) The frequency of false positioning without applying the CP mechanism. (**b**) The frequency of false positioning when applying the CP mechanism. (**c**) The impact of the *ε* to the frequency of false positioning when applying the CP mechanism. Increasing the *ε* can lower the frequency of the false positioning.

Next, the signal irregularity is considered, and the number of false positioning results is evaluated. False positioning means that the target is not enclosed within the positioning area. In this experiment, the ratio of the signal irregularity to radio range is used to simplify complexity channel interferences caused by the environmental factors. The worst case of signal irregularity is considered to be the comparison. Figure 8 shows the number of false positioning results for 1000 different topologies. The experimental results without the CP mechanism are depicted in Figure 8a, and the results with the CP mechanism are shown in Figure 8b. The radio range in this experiment is 20 m. The degree of signal irregularity (DoI) rates being evaluated, denoted by *p*, are {0.2, 0.15, 0.1, 0.05}. Here, *p* for the DoI indicates that the sensor node has a 50% probability of failing to detect the target when the target is in its *uReg*. The presence of the target in the *uReg* of a sensor node means that the distance from target to the sensor node is between *R* × (1 − *p*) and *R*. For example, DoI = 0.05 indicates that the target is within the *uReg* of the sensor node, and its distance to the sensor node is between 19 m and 20 m.

The number of false positioning results occurs as the number of sensor nodes increases. Sensors within the *uReg* may contribute a fault detecting information to the sink. Thus, when more sensor nodes are deployed, the probability that sensor nodes appear within the *uReg* increases. This causes the number of false positioning grows. Figure 8a shows the results without applying the CP mechanism. The number of false positioning results is more than 300, even in the case with 30 sensor nodes and a low degree *p* = 0.05. When 65 sensor nodes are deployed, the number of false positioning results is around 750, which comprises approximately three-quarters of all simulation topologies. When a high degree *p* = 0.2 is employed, 450 and 920 false positioning results were obtained in the cases with 30 and 65 sensor nodes, respectively.

By applying the CP mechanism, the number of false positioning results can be significantly reduced, and is no more than 120 in the case with 30 sensor nodes for any degree *p*. Furthermore, the number of false positioning results was less than 270 in the case with 65 sensor nodes. This result represents around a quarter of that without applying the CP mechanism. This proves that the CP mechanism is effective in reducing the number of false positioning results in a real scenario. The values of the corresponding confidence interval of Figure 8a,b are given in Table 3 and Table 4. The confidence interval in the case of *p* = 0.2 is larger than the case of *p* = 0.05. The scenario with higher DoI generates more oscillation in the results because more sensor nodes may provide false information on positioning.

Finally, different size of ε is applied to evaluate the convergence rate of the frequency of the false positioning shown as Figure 8c. Increasing the size of ε can reduce the frequency of false positioning. When the 1.5ε is applied, the number of false positioning is fewer than 80 that is smaller than 10% of the total 1000 simulation times. As the 1.75ε is applied, the ratio of false positioning is less than 3% of the total simulation times. Increasing the size of ε indicates more sensor nodes near the uncertainty region will be removed. The sensor nodes that may provide false information of the target can be expelled so that the frequency of false positioning can be reduced. On the contrary, the target-undetected sensor nodes that can be used to prune the positioning area are also removed. Thus, the positioning accuracy will be sacrificed.

**Figure 9:**
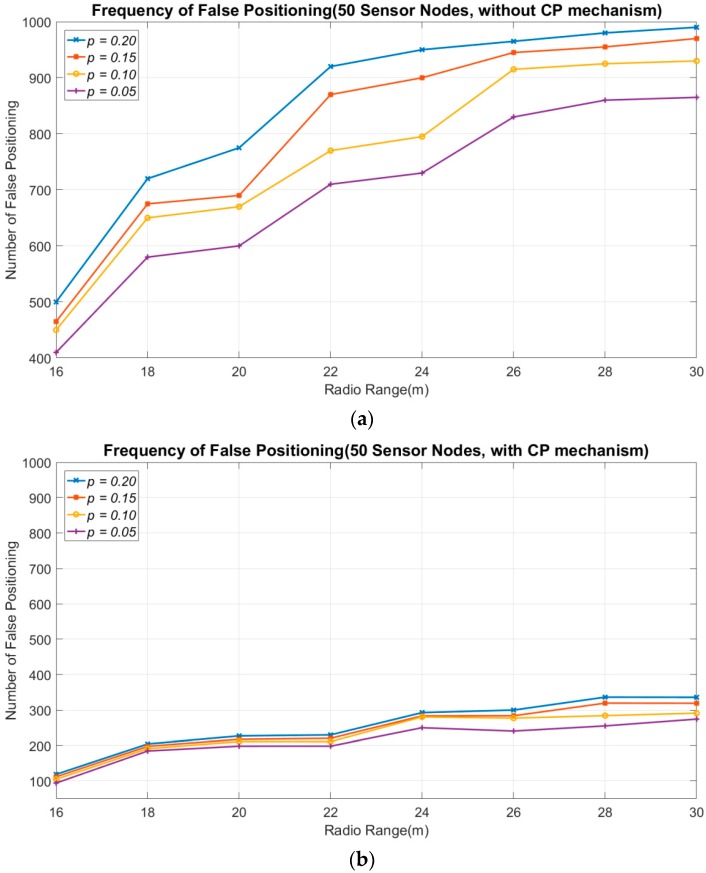
The frequency of false positioning vs. the radio range (*p* is the degree of signal irregularity). (**a**) The frequency of false positioning without applying the CP mechanism. (**b**) The frequency of false positioning when applying the CP mechanism.

We also evaluated the impact of the radio range, shown in Figure 9. The number of deployed sensor nodes is 50, and the radio range varies between 16 m and 30 m. A short radio range can reduce the probability of false positioning, because the introduced *uReg* is small. In the case when the radio range is 16 m, the number of false positioning results is approximately 400–500 without the CP mechanism. However, the number of false positioning results is greater than 800 when the radio range is above 25 m. In the case of a high degree *p* = 0.2, the number of false positioning results is more than 98%, as shown in Figure 9a. By applying the CP mechanism, the number of false positioning results with a radio range of 16 m becomes smaller than 125. The worst number of false positioning results in the radio range of 30 m is no more than 340, as shown in Figure 9b. The values of the corresponding confidence interval of Figure 9a,b are given in Table 5 and Table 6. Like in Figure 8, the confidence interval in the case of higher DoI generates more oscillation.

## 5. Conclusions

A novel range-free positioning system called CAPPS was proposed in this study. CAPPS first employs the sensor nodes that can detect the target to obtain an approximate location of the target. Then, this area is pruned by using the sensor nodes that cannot detect the target. To reduce the computation time, CAPPS refines the set of sensor nodes used to prune the positioning area. Considering the application to a practical scenario, the signal irregularity was also evaluated, and a CP mechanism was proposed to resolve the false positioning problem. Simulation results demonstrated that CAPPS achieves a higher positioning accuracy than the DV-Hop, AoA, and RSSI methods. The rate of improvement to the size of the positioning area is greater than 93%. In the irregular signal scenario, applying the CP mechanism can efficiently reduce the number of false positioning results from 50%–95% to 10%–30%.

## Figures and Tables

**Table 1:** Simulation Parameters.

Simulation Parameters	Value
Size of RoI	100 × 100 m^2^
Shape of radio signal	Perfect disk
The precision of an AoA node	15° (±7.5°).
Directional antenna of an AoA node	2
Signal-to-distance transfer error in RSSI	0%–15%
Simulation attempts	2000 times
Number of deployed sensor nodes	30–65
Radio range of each sensor node	15–30 m

**Table 2:** The numerical results of the positioning area size (m^2^) in Figure 4a.

	Number of Nodes	30	35	40	45	50	55	60	65
Methods									
CAPPS		47	35	25	20	18	15	12	10
RSSI		140	138	137	138	136	139	137	138
AoA		385	390	386	384	390	386	378	382
DV-Hop		608	614	612	622	630	636	634	632

**Table 3:** The confidence interval of Figure 8a.

	Number of Nodes	30	35	40	45	50	55	60	65
*p*									
0.2		±9.23	±10.98	±13.28	±15.09	±17.75	±20.54	±23.38	±26.09
0.15		±6.10	±7.92	±8.75	±11.23	±12.68	±14.03	±15.78	±16.73
0.1		±4.72	±5.80	±6.17	±7.41	±8.87	±10.30	±11.42	±13.17
0.05		±3.53	±4.76	±5.74	±6.76	±8.18	±9.09	±10.41	±11.46

**Table 4:** The confidence interval of Figure 8b.

	Number of Nodes	30	35	40	45	50	55	60	65
*p*									
0.2		±3.68	±5.75	±6.62	±7.80	±9.19	±10.13	±10.61	±11.31
0.15		±2.99	±3.85	±4.79	±5.69	±5.98	±6.95	±7.58	±8.46
0.1		±2.23	±3.17	±3.75	±4.58	±4.97	±5.53	±6.20	±6.95
0.05		±2.02	±2.74	±3.43	±4.16	±4.49	±4.98	±5.81	±5.98

**Table 5:** The confidence interval of Figure 9a.

	Number of Nodes	30	35	40	45	50	55	60	65
*p*									
0.2		±11.99	±15.88	±18.51	±20.93	±21.73	±22.88	±24.15	±24.12
0.15		±8.18	±12.24	±13.31	±15.16	±16.70	±17.15	±17.35	±18.19
0.1		±6.55	±9.87	±10.67	±11.57	±12.46	±13.52	±14.29	±14.90
0.05		±5.95	±8.09	±9.29	±10.95	±11.80	±13.53	±13.70	±13.99

**Table 6:** The confidence interval of Figure 9b.

	Number of Nodes	30	35	40	45	50	55	60	65
*p*									
0.2		±2.61	±2.45	±5.16	±5.35	±6.37	±6.57	±7.79	±8.24
0.15		±2.06	±3.48	±3.93	±4.12	±5.09	±5.24	±5.75	±5.92
0.1		±1.68	±2.93	±3.27	±3.5	±4.17	±4.44	±4.53	±4.53
0.05		±1.36	±2.9	±3.1	±3.35	±4.12	±4.08	±4.17	±4.42

## References

[1] Yin S., Liu L., Zhou R., Sun S., Wei S. (2013). Design of wireless multi-media sensor network for precision agriculture. China Commun..

[2] Stattner E., Vidot N., Hunel P., Collardens M., McLeod B. Wireless sensor network for habitat monitoring: A counting heuristic. Proceedings of the IEEE 37th Local Computer Networks Workshops.

[3] Tovar A., Friesen T., Ferens K., McLeod B. A DTN wireless sensor network for wildlife habitat monitoring. Proceedings of the 23rd Canadian Conference on Electrical and Computer Engineering (CCECE).

[4] Yang H., He Y. Wireless Sensor Network for Orchard Soil and Climate Monitoring. Proceedings of the World Congress on Computer Science and Information Engineering.

[5] Ferre J.A., Pawlowski A., Guzman J.L., Rodriguez F., Berenguel M. A Wireless Sensor Network for greenhouse climate monitoring. Proceedings of the Fifth International Conference on Broadband and Biomedical Communications (IB2Com).

[6] Jain A.K., Khare A., Pandey K.K. Developing an efficient framework for real time monitoring of forest fire using wireless sensor network. Proceedings of the 2nd IEEE International Conference on Parallel Distributed and Grid Computing (PDGC).

[7] Zhu Y., Xie L., Yuan T. Monitoring system for forest fire based on wireless sensor network. Proceedings of the 10th World Congress on Intelligent Control and Automation (WCICA).

[8] Rahman M.N., Hanuranto M.T.I.A.T., Mayasari S.T.M.T.R. Trilateration and iterative multilateration algorithm for localization schemes on Wireless Sensor Network. Proceedings of the 2017 International Conference on Control Electronics Renewable Energy and Communications (ICCREC).

[9] Zhang G.J., Li X., Xu Z.L., Li H.C. Weighted Least Square Localization Algorithm Based on RSSI Values. Proceedings of the Fifth International Conference on Instrumentation and Measurement, Computer, Communication and Control (IMCCC).

[10] Tomic S., Beko M., Dinis R., Lipovac V. RSS-based localization in wireless sensor networks using SOCP relaxation. Proceedings of the IEEE 14th Workshop on Signal Processing Advances in Wireless Communications (SPAWC).

[11] Tomic S., Beko M., Dinis R. (2014). Distributed RSS-Based Localization in Wireless Sensor Networks Based on Second-Order Cone Programming. Sensors.

[12] Tomic S., Beko M., Dinis R. (2015). RSS-Based Localization in Wireless Sensor Networks Using Convex Relaxation: Noncooperative and Cooperative Schemes. IEEE Trans. Veh. Technol..

[13] Gottapu S.K., Vallabhaneni P. Wireless Sensor Network Localization in 3D using Steerable Anchors’ Antennas. Proceedings of the 2018 International Conference on Signal Processing and Communication Engineering Systems (SPACES).

[14] Ennsar O., Xing G., Tan X. Distributed time-difference-of-arrival (TDOA)-based localization of a moving target. Proceedings of the 55th International Conference on Decision and Control (CDC).

[15] Jin B., Xu X., Zhang T. (2018). Robust time-difference-of-arrival (TDOA) localization using weighted least squares with cone tangent plane constraint. Sensors.

[16] Yao L., Dai Z., Yang S., Ding H. A quadratic centroid algorithm for wireless sensor network localization. Proceedings of the 36th Chinese Control Conference (CCC).

[17] Shu Y., Cheng P., Gu Y., Chen J., He T. Toc: Localizing wireless rechargeable sensors with time of charge. Proceedings of the 2014 Conferences on INFOCOM.

[18] Anthrayose S., Payal A. Comparative Analysis of Approximate Point in Triangulation and DV-Hop Algorithms for Solving Localization Problem in Wireless Sensor Networks. Proceedings of the Seventh International Conference on Advance Computing Conference.

[19] Cheikhrouhou O., Bhatti G.M., Alroobaea R. (2018). A Hybrid DV-Hop Algorithm Using RSSI for Localization in Large-Scale Wireless Sensor Networks. Sensors.

[20] Wang Z., Zhang B., Wang X., Jin X., Bai Y. (2018). Improvements of Multihop Localization Algorithm for Wireless Sensor Networks. IEEE Syst. J. (Early Access).

[21] Wang J.Z., Jin H. Improvement on APIT Localization Algorithms for Wireless Sensor Networks. Proceedings of the 2009 International Conference on Networks Security, Wireless Communications and Trusted Computing.

[22] Sharma R., Malhotra S. (2015). Approximate Point in Triangulation (APIT) based Localization Algorithm in Wireless Sensor Network. Int. J. Innov. Res. Sci. Technol..

[23] Badawy A., Khattab T., Trinchero D., ElFouly T., Mohamed A. A Simple Angle of Arrival Estimation System. Proceedings of the 2017 IEEE Wireless Communications and Networking Conference (WCNC).

[24] Tomic S., Beko M., Dinis R., Bernardo L. (2018). On Target Localization Using Combined RSS and AoA Measurements. Sensors.

[25] Gupta P., Verma V.K., Senapati V. Angle of arrival detection by ESPRIT method. Proceedings of the 2017 International Conference on Communication and Signal Processing (ICCSP).

[26] Huang S.C. (2017). The Novel Positioning System based on Coverage Elimination in Wireless Sensor Networks. Int. J. Adv. Comput. Eng. Netw..

[27] Chang Z., Wu X., Wang W. Localization in Wireless Rechargeable Sensor Networks Using Mobile Directional Charger. Proceedings of the Global Communications Conference (GLOBECOM).

[28] He T., Huang C.D., Blum B.M., Stankovic J.A., Abdelzaher T. Range-Free Localization Schemes for Large Scale Sensor Networks. Proceedings of the 9th annual international conference on Mobile computing and networking.

[29] Yuan B., Su Z., Lu H.F., Shu B. The optimization research of node localization algorithm based on wireless sensor network. Proceedings of the 7th International Conference on Biomedical Engineering and Informatics.

[30] Huang S.C. (2011). Ion-6: A Positionless Self-Deploying Method for Wireless Sensor Networks. Int. J. Distrib. Sens. Netw..

